# SeqFu: A Suite of Utilities for the Robust and Reproducible Manipulation of Sequence Files

**DOI:** 10.3390/bioengineering8050059

**Published:** 2021-05-07

**Authors:** Andrea Telatin, Piero Fariselli, Giovanni Birolo

**Affiliations:** 1 Gut Microbes and Health Programme, Quadram Institute Bioscience, Norwich NR4 7UQ, UK; andrea.telatin@quadram.ac.uk; 2 Department of Medical Sciences, University of Turin, 10126 Torino, Italy; giovanni.birolo@unito.it

**Keywords:** bioinformatics, FASTQ, FASTA, software, next-generation sequencing

## Abstract

Sequence files formats (FASTA and FASTQ) are commonly used in bioinformatics, molecular biology and biochemistry. With the advent of next-generation sequencing (NGS) technologies, the number of FASTQ datasets produced and analyzed has grown exponentially, urging the development of dedicated software to handle, parse, and manipulate such files efficiently. Several bioinformatics packages are available to filter and manipulate FASTA and FASTQ files, yet some essential tasks remain poorly supported, leaving gaps that any workflow analysis of NGS datasets must fill with custom scripts. This can introduce harmful variability and performance bottlenecks in pivotal steps. Here we present a suite of tools, called SeqFu (Sequence Fastx utilities), that provides a broad range of commands to perform both common and specialist operations with ease and is designed to be easily implemented in high-performance analytical pipelines. SeqFu includes high-performance implementation of algorithms to interleave and deinterleave FASTQ files, merge Illumina lanes, and perform various quality controls (identification of degenerate primers, analysis of length statistics, extraction of portions of the datasets). SeqFu dereplicates sequences from multiple files keeping track of their provenance. SeqFu is developed in Nim for high-performance processing, is freely available, and can be installed with the popular package manager Miniconda.

## 1. Introduction

The FASTA format was introduced in 1985 with the homonym software package developed by Lipman and Pearson [1]. It is still the *de facto* standard format for nucleotide and protein sequences. With the advent of automatic capillary sequencing, the FASTQ format was introduced to store a quality score for each base [1,2]. These two file formats are ubiquitous in bioinformatics, and a broad set of utilities have been released over the years to help the users access and manipulate the sequences (from filtering tools like Cutadapt [3] and Fastp [4], to general toolkits like SeqKit [5] and SeqTk [6]).

Here we present SeqFu, a novel suite of utilities to manipulate FASTA and FASTQ files. It is written in the Nim programming language which combines an intuitive syntax inspired by Python with the performance (and ease of distribution) of compiled programs. 

SeqFu provides easy access to commonly used operations (e.g., interleaving/deinterleaving FASTQ files), tools to facilitate testing and manual troubleshooting (e.g., inspecting FASTQ files and checking for the presence of oligonucleotide sequences), and more specialized utilities. 

Generic templates to prototype *ad hoc* tools are also available from the software repository.

## 2. Materials and Methods

SeqFu is written in Nim, a high-performance compiled language, was tested using three compiler versions (1.0, 1.2, and 1.4), and implements the FASTA/FASTQ parsing algorithm written by Heng Li [7], which is available from the repository https://github.com/lh3/biofast/ (accessed on 12 April 2021). SeqFu was designed and tested to run on Linux and macOS, but it should be possible to compile it for other POSIX compliant systems. 

Some modules and templates use an alternative implementation of the same algorithm called readfq (https://github.com/andreas-wilm/nimreadfq, accessed on 12 April 2021), while *k*-mer counting is achieved using the implementation given by the nimbioseq library (https://github.com/jhbadger/nimbioseq, accessed on 12 April 2021).

The Perl library (FASTX::Reader), which implements the same algorithm for FASTX file parsing, is platform-independent and requires Perl 5.12 or greater.

The full SeqFu code is freely available from the GitHub repository (https://github.com/telatin/seqfu2, accessed on 12 April 2021), and its documentation is available at https://telatin.github.io/seqfu2 (accessed on 12 April 2021).

The suite is automatically checked at each release by a set of tests.

## 3. Results

SeqFu consists of a set of core modules bundled in a main ‘seqfu’ executable and an additional set of corollary utilities, each in a separate binary executable with the ‘fu-’ prefix.

The FASTQ/FASTA parsing library we adopted allows FASTA or FASTQ files to be used as input files, compressed with or without *gzip*, and includes support for the less common ‘Sanger FASTQ’ format that allowed a single sequence to span multiple lines.

The algorithm to scan for sub-sequences in a sequence string allows the query to be inserted using IUPAC ambiguous bases and will return full or partial matches from both strands of the target sequence.

When paired-end input files are expected, the programs only specify the first pair file name, autodetecting the paired reverse.

### 3.1. Manual Inspection of Datasets

SeqFu facilitates the testing of pipelines and workflows and has tools to inspect input files and view or extract the first or last sequences of a dataset. 

**Figure 1:**
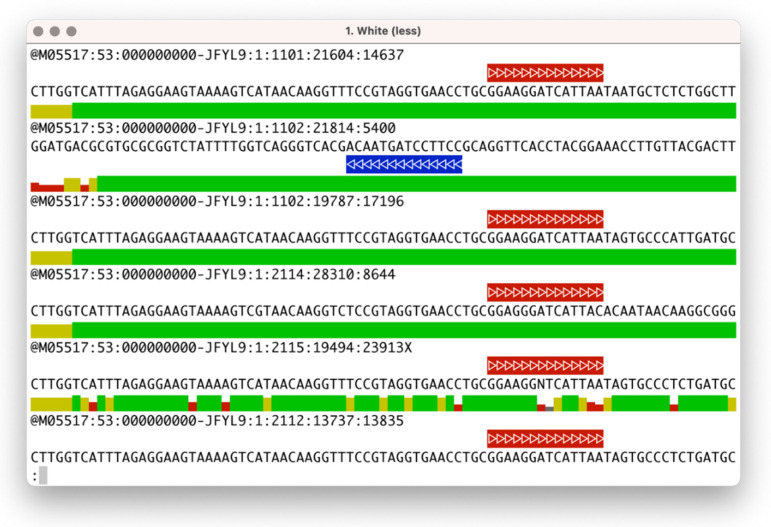
Example of the rendering of a FASTQ file by *seqfu view*, where the quality track is represented by a block of variable height and colors (gray, red, yellow, and green) and oligonucleotide matches are rendered as colored arrows.

The *seqfu view* subprogram will render the quality string in the form of a coloured bar plot using Unicode characters and includes an ‘oligonucleotide match’ function to visually highlight the presence of primers, adaptors, or other sequences of interest in the input file. This utility can be helpful for performing a first manual inspection of datasets or evaluating the efficacy of trimming steps and inspecting their output files. A screenshot of *seqfu view* with oligonucleotide matches highlighted is shown in Figure 1.

The *seqfu head* and *seqfu tail* functions allow extraction of the first (or last) sequences from a dataset, mimicking the commonly used *head* and *tail* Unix utilities, with the addition of parameters to skip a number of sequences allowing, for example, the user to extract one sequence every 12, limited to the first 100 occurrences.

Similarly, *seqfu grep* mimics the ‘GNU grep’ utility but allows for queries, both in the sequence name (either exact matches or regular expressions), and in the sequence itself. In the latter case, the oligonucleotide match is achieved as described above, allowing both strands to be queried and setting thresholds to allow for partial matches.

A first overview of a FASTQ dataset can be obtained with *seqfu qual*, that will report the inferred quality encoding (e.g., ‘Illumina 1.8’) and an average quality profile of the reads.

### 3.2. FASTQ Dataset Management

There are routine operations on FASTQ datasets (and specifically Illumina paired-end FASTQ datasets) that are commonly performed using Bash scripts, but which would definitely benefit from a more robust implementation with consistency checks and better performance and reliability from the unit tests.

Paired-end sequences can be stored in two separate files or in a single ‘interleaved’ file, where its paired reverse sequence follows each forward sequence. SeqFu implements the *interleave* and *deinterleave* functions that convert separate pairs into a single interleaved file or split an interleaved file into separate pairs, respectively. 

Another commonly performed task is merging the FASTQ files coming from different lanes of some Illumina sequencers. This task can be achieved with a *seqfu lanes* function, which processes a full run with a single command that requires half the time needed for a popular Bash script to achieve the same operation (see Section 3.7).

### 3.3. Sequence Statistics

A common operation, also performed by other tools like SeqKit, is counting the number of sequences in one or more files. *Seqfu count* performs this operation for all supplied input files. It still detects paired-end datasets and prints the reads count only once per paired dataset, ensuring that both files have the same number of sequences; if the numbers are not the same, then an error is reported.

**Figure 2:**
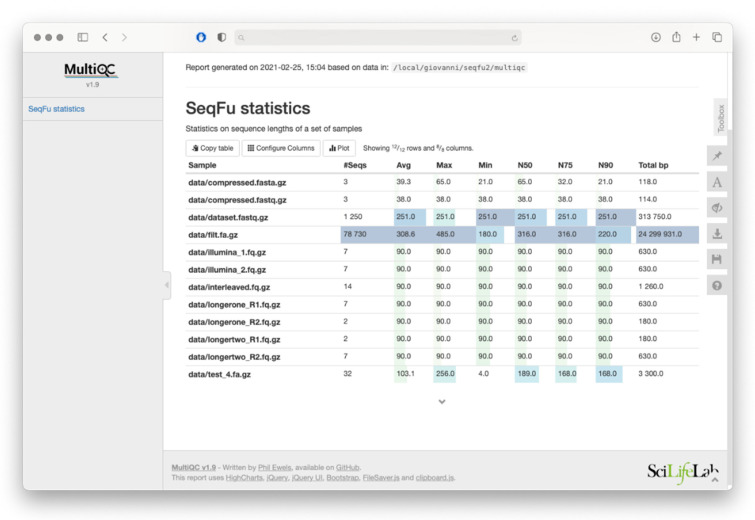
MultiQC report generated by *seqfu stats*. Furthermore, *seqfu counts* can generate a similar table.

The *seqfu stats* utility calculates a set of statistics based on the length of sequences in the input files (including the widely adopted *N50*, and the less commonly used *auN* [8,9]). It offers the option to print the output as raw tables, screen-friendly tables, or as a MultiQC-ready file that can be incorporated with ease into a report generated by the MultiQC tool [10]. An example of a MultiQC report generated with *seqfu stats* is shown in Figure 2.

Seqkit provides a similar function, so we compared the performance of *seqfu stats* with *seqkit stat* (see Section 3.8), and found an improvement in speed despite the additional calculations performed.

### 3.4. Other Utilities

*Seqfu derep* allows the dereplication of FASTA and FASTQ datasets, removing duplicate sequences and keeping track of the total number of occurrences found in the original dataset. SeqFu adds some user-friendly features compared with other tools (e.g., VSEARCH [11]), such as the possibility of propagating the number of identical sequences initially present in a file. 

This means that, if a pipeline requires multiple dereplication steps, it remains possible to identify the initial number of sequences of a specific type; this is because the information is passed at each step via the ‘size = NUMBER’ tag in the sequence header. The program can also generate a report, in JSON format, on the origin of the duplicated reads (to identify which files contained each sequence and how many times per file). 

These features, missing in alternative packages, can provide the foundation for performance improvements in metagenomics and metabarcoding pipelines.

The tool *fu-orf* allows the extraction of open reading frames (ORFs) from nucleotide datasets, including paired-end reads that are joined before ORF extraction. The module translates each input sequence using the standard genetic code and will return all the open reading frames (that can be filtered requiring a minimum length).

Other utilities include *seqfu sort* (to sort sequences by size), *fu-sw* (to perform a local alignment against a target sequence using the Smith-Waterman algorithm [12]), *seqfu rc* (to reverse complement sequences, supporting degenerate bases written as IUPAC DNA characters), and *fu-primers* (to mask degenerate primers from FASTQ files).

### 3.5. Generic FASTX Utility Templates

The software repository contains a set of templates for custom application based on FASTA or FASTQ file parsing. When each sequence is processed in an independent task, the process can be engineered in a multithreading application, for which we also provide specific templates.

### 3.6. Perl Library with The FASTX Parser

The FASTA/FASTQ parser used in SeqFu is also available as a Perl module (FASTX::Reader), that can be found in MetaCPAN (https://metacpan.org/release/FASTX-Reader, accessed on 20 April 2021) and BioConda (as ‘perl-fastx-reader’).

### 3.7. SeqFu Performance of Interleave, Deinterleave

We evaluated the SeqFu performance of *interleave, deinterleave*, and *lanes* programs were compared with commonly used Bash one-liners (that usually lack any control of input and output integrity). To make the comparison more relevant, we used uncompressed FASTQ files and restricted the commands to a single core. These three subprograms were implemented in SeqFu using a single thread.

**Figure 3:**
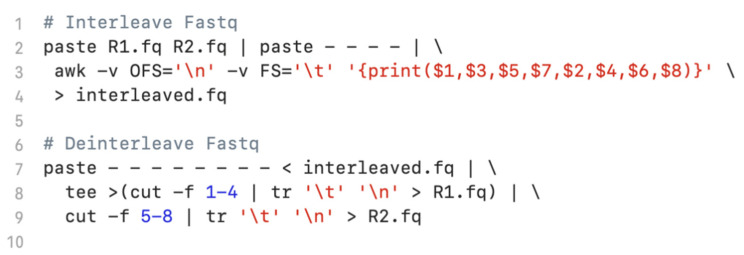
Bash script commonly used to interleave and deinterleave FASTQ files. At line 2 the command used to interleave two FASTQ files; at line 7 the command used to deinterleave a FASTQ file.

We compared *seqfu interleave* and *seqfu deinterleave* with the Bash commands shown in Figure 3.

SeqFu was two times faster than Bash for interleaving, taking 4.7 ± 0.14 s versus 11.4 ± 0.07 s and three times faster for deinterleaving, taking 3.9 ± 0.08 s versus 12.8 ± 0.10 s. It should be noted that SeqFu provides easier access to the functions and a set of tests to prevent reading-corrupted input files or producing invalid output. 

The *seqfu lanes* program has been compared with a popular Bash script available via GitHub (https://github.com/stephenturner/mergelanes, accessed on 1 May 2021), which benefits from the multithreading capabilities of Bash pipes, but does not perform any integrity check on the input files, with the possibility of producing corrupted datasets. We detected a 10-fold increase in speed (2.6 ± 0.9 ms for *seqfu lanes* compared with 31.8 ± 4.0 ms for the Bash script).

### 3.8. Comparison between SeqFu and SeqKit 

We compared the performance of the *stats* module with two other utilities: ‘SeqKit’ and ‘n50’, both available from BioConda. We used a file with only a few large sequences (the human genome reference) and another file with many small sequences (reference bacterial genomes of the gastrointestinal tract from the NIH Human Microbiome Project), both used to benchmark SeqKit (https://bioinf.shenwei.me/seqkit/benchmark/, accessed on 28 April 2021). Both datasets account for ~3 Gbp, but the Human genome reference is composed of 194 sequences, while the other is more fragmented with 67,748 sequences.

**Figure 4:**
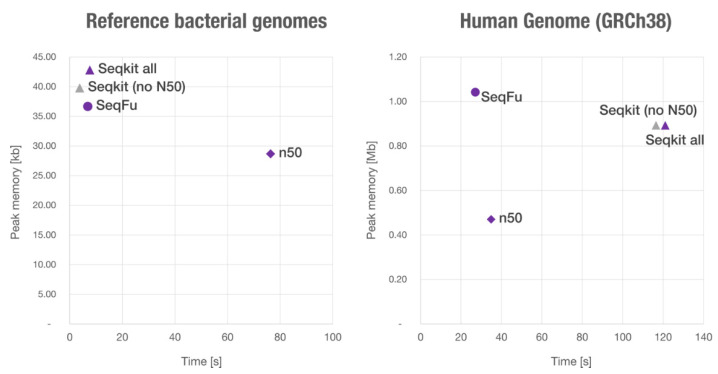
Memory consumption and execution times of Seqkit, SeqFu and n50 using two datasets (reference bacterial genomes from the gastrointestinal tract, left, and the human genome, right). SeqKit has been used with default parameters (no N50) which skips the calculation of extended statistics, and with the ‘—all’ parameter (labelled as *Seqkit all*). SeqFu is the faster tool in both datasets, with a remarkable difference when parsing a file with few large sequences (human genome).

We used ‘*seqfu stats*’, ‘*seqkit stats --all*’ and ‘*n50 -x*’ to ensure a similar output (by default, *SeqKit* does not calculate the N50), finding that SeqFu is four times faster with the human genome, and 1.1 times faster with the other dataset (thus the programs have similar performance with many sequences). We also compared the (peak) memory usage, which is similar for the considered programs (~1 Mb for the human genome, ~40 kb for the short sequences), and is determined by the size of the largest sequence parsed (see Figure 4).

## 4. Discussion

In bioinformatics, it is currently possible and relatively easy to find software to perform complex tasks (such as sequence alignment, variant calling, genomic assembly). Many choices are available, and the community converges towards well-performing, high-quality and thoroughly-tested solutions. The situation is different for simpler tasks: for many of these the available solutions are few and often lacking in some way, since there is little merit in publishing these type of tools. Thus, it is common to rely on home-made scripts, bash hacks, and ‘glue’ code, resulting in slow, hard-to-use, and untested applications. 

SeqFu aims to improve this situation by providing an easily deployable set of well-documented, tested, and high-performance utilities. The recommended deployment strategy is through the widely adopted ‘Miniconda’ package manager (and specifically via the ‘BioConda’ channel [11]), while the code is published on GitHub. Full documentation is available online and in a compact format in the built-in command help. 

High-performance follows from using a compiled language, which is relevant for a tool to be used on real data. Since we focused on features unavailable in other tools, it is difficult to perform a comprehensive performance comparison due to the lack of alternative implementations. The common tasks of interleaving and deinterleaving paired-end reads, for example, are often performed with Bash scripts that are harder to implement, more error-prone, but also less efficient than SeqFu, which is up to three times faster. Similarly, the common operation of merging reads from multiple lanes of Illumina sequencers is ten times faster than the implementation provided by a popular Bash script. SeqFu stats is a subcommand that has a direct alternative in the well-known SeqKit tool. However, SeqFu is up to four times faster than SeqKit on datasets with large sequences.

The provided commands are reasonably generic, providing many options to customize the results. However, by design, we keep the scope of each command limited to simple commands for simple tasks.

In addition to common tasks, there are several scenarios where *ad hoc* scripts are required to perform operations tailored for custom sequencing libraries or downstream applications. It is common practice to use high-end programming languages like Python for these kinds of tasks, given the simple syntax and excellent parsing libraries available. We believe Nim to be an equally accessible language (thanks to a Python-inspired syntax) that can bring a performance boost in these scenarios (being a compiled language), hence our decision to curate a set of program templates that are easy to compile and distribute.

## 5. Conclusions

The SeqFu suite is an easy-to-install set of tools to manipulate FASTA and FASTQ files that fills some gaps left by other software in this category. SeqFu offers templates to build custom programs that can manipulate sequencing datasets with the performance advantage offered by a compiled language and multithreading support.

Finally, SeqFu provides novel approaches for high performance processing of NGS datasets, like an improved dereplication tool (that can be particularly beneficial in metagenomics and metabarcoding pipelines), and simplify the reporting of its results both via JSON and MultiQC-enabled output.

## Data Availability

The published version of the software has been archived in Zenodo, and available at https://doi.org/10.5281/zenodo.4740106 (accessed on 6 May 2021).
